# Efficient Photoacoustic Imaging With Biomimetic Mesoporous Silica-Based Nanoparticles

**DOI:** 10.3389/fbioe.2021.762956

**Published:** 2021-11-30

**Authors:** Chuangjia Huang, Xiaoling Guan, Hui Lin, Lu Liang, Yingling Miao, Yueheng Wu, Huiqiong Bao, Xiaodan Wu, Ao Shen, Minyan Wei, Jionghua Huang

**Affiliations:** ^1^ Department of Cardiology, The Third Affiliated Hospital, Guangzhou Medical University, Guangzhou, China; ^2^ Key Laboratory of Molecular Target and Clinical Pharmacology and the State and NMPA Key Laboratory of Respiratory Disease, School of Pharmaceutical Sciences, The Fifth Affiliated Hospital, Guangzhou Medical University, Guangzhou, China; ^3^ Department of Oncology, Guangdong Provincial Hospital of Integrated Traditional Chinese and Western Medicine, Foshan, China; ^4^ School of Medicine, Guangdong Provincial People’s Hospital and Guangdong Academy of Medical Sciences, South China University of Technology, Guangzhou, China

**Keywords:** ICG, mesoporous silica nanoparticles, cell membrane coating, photoacoustic imaging, cervical carcinoma

## Abstract

Indocyanine green (ICG), a near-infrared (NIR) fluorescent dye approved by the Food and Drug Administration (FDA), has been extensively used as a photoacoustic (PA) probe for PA imaging. However, its practical application is limited by poor photostability in water, rapid body clearance, and non-specificity. Herein, we fabricated a novel biomimetic nanoprobe by coating ICG-loaded mesoporous silica nanoparticles with the cancer cell membrane (namely, CMI) for PA imaging. This probe exhibited good dispersion, large loading efficiency, good biocompatibility, and homologous targeting ability to Hela cells *in vitro*. Furthermore, the *in vivo* and *ex vivo* PA imaging on Hela tumor-bearing nude mice demonstrated that CMI could accumulate in tumor tissue and display a superior PA imaging efficacy compared with free ICG. All these results demonstrated that CMI might be a promising contrast agent for PA imaging of cervical carcinoma.

## 1 Introduction

Cervical carcinoma has become the fourth most prevalent malignant cancer in women, affecting nearly 600, 000 women worldwide annually (Koh et al., 2019; Arbyn et al., 2020). And cervical carcinoma is also the fourth leading cause of cancer-related death in women, killing approximately 300,000 women globally every year (Koh et al., 2019; Arbyn et al., 2020). Early detection and precise diagnosis are significantly important to effective treatment of cervical carcinoma (Huang et al., 2021; Koh et al., 2019). Photoacoustic (PA) imaging is a non-invasive and non-ionizing biomedical imaging modality which is combined with optical excitation and ultrasonic detection (Zeng et al., 2018). PA imaging offers high resolution, rich contrast, deep tissue penetration, and lack of irradiation, which has received enormous attention in cancer detection (Sun et al., 2019a; Qiu et al., 2021). The near-infrared (NIR) fluorescence used as PA probes for PA imaging could obtain a strong PA signal due to high photo penetration depth and low background autofluorescence, but its tumor-targeting specificity should be improved (Meng et al., 2018; Reinhardt and Chan, 2018; Sun et al., 2019b). Therefore, the tumor-targeting specificity remains a challenge for the PA imaging of cancer.

Indocyanine green (ICG), which is a near-infrared (NIR) fluorescent dye approved by the Food and Drug Administration (FDA), has been extensively used as a photoacoustic (PA) probe for PA imaging (Reinhardt and Chan, 2018; Xu et al., 2019). However, its practical application is limited by poor photostability in water, rapid body clearance, and non-specific tumor targeting (Gao et al., 2018). To overcome these limitations, extensive research studies have been conducted to encapsulate ICG in various organic nanocarriers, including liposomes, polymeric micelles, and polymer nanoparticles. Nevertheless, its poor physicochemical instability and serum-induced drug leakage are still the main challenges for ICG delivery and PA imaging (Huang et al., 2020).

Mesoporous silica nanoparticles (MSNs), categorized as “generally regarded as safe” materials by the FDA, have attracted great attention on drug and fluorescent dye delivery for cancer diagnosis and therapy (Chaudhary et al., 2019; Gao et al., 2020). Due to the unique mesoporous structure, MSNs not only display huge surface area, large pore volume and tunable pore diameter in physical characteristics but also exhibit good biocompatibility and excellent passive targeting ability in biological behavior (Wang et al., 2015; Jafari et al., 2019). MSNs with large pore volume could provide great potential for the drug payload without premature release, which might show better physical stability and less drug leakage than other nanocarriers (Croissant et al., 2016; Jafari et al., 2019). Furthermore, the surface of MSNs could be modified with either artificial materials or natural substances, which could improve the long circulation characteristics and active tumor-targeting ability of MSNs (Li et al., 2020). Functionalized MSNs were utilized for ICG loading to improve its photostability and body clearance (Chaudhary et al., 2019). Therefore, MSNs might be a promising approach for ICG loading and its application for PA imaging.

Cell membrane coating is widely used to fabricate biomimetic nanoparticles, which has been considered as a promising approach for precise tumor diagnosis and treatment (Fang et al., 2018; Zhang L. et al., 2020; Zhang Y. et al., 2020; Li et al., 2020). Various cell types, including red blood cells (RBC) (Dehaini et al., 2017; Kim et al., 2020), platelets (Dehaini et al., 2017; Kunde and Wairkar, 2021), white blood cells (Yaman et al., 2020), cancer cells (Yang et al., 2018) and stem cells (Defterali et al., 2016), have been coated with the nanoparticles (Fang et al., 2018). In comparison with polymer coating, cell membranes coated with nanoparticles exhibited low immunogenicity and excellent homologous targeting ability (Fang et al., 2018; Li et al., 2020; Lin et al., 2021; Peng et al., 2021). For cancer diagnosis and treatment, based on the homologous targeting ability, nanoparticles coated with the cancer cell membrane from homologous cells could efficiently deliver drugs and probes to the tumors (Fang et al., 2018; Zhang L. et al., 2020). Therefore, MSNs fabricated with the cancer cell membrane from the homologous Hela cells might efficiently deliver ICG to the cervical tumor and significantly enhance the PA imaging for cervical cancer *via* the homologous targeting ability.

In this study, a novel biomimetic nanoprobe with ICG-loaded mesoporous silica nanoparticles modified with the cancer cell membrane (CCM/MSNs@ICG, CMI) was fabricated, and its homologous targeting ability and PA imaging effect on cervical carcinoma were also investigated. MSNs were prepared by the sol–gel method. ICG was loaded into the pores of MSNs, and the surface of MSNs was modified with the cancer cell membrane of Hela cells to improve their biocompatibility and homologous targeting ability. Then, the CMI was characterized in detail. In order to evaluate the homologous targeting ability of the cancer cell membrane–coated MSNs, DiD was chosen as a model fluorescent dye, and Hela cellular uptakes of CMD were observed by using a confocal microscope. To confirm the biocompatibility of the CMI, an *in vitro* cytotoxicity study was carried out by calcein-AM/PI staining and CCK-8 assay. Finally, in order to investigate the PA imaging efficacy of the CMI, *in vivo* and *ex vivo* PA imaging were further carried out on the Hela tumor-bearing mice. The novel nanoprobe might increase the ICG accumulation on the tumor and enhance the PA imaging efficacy of cervical carcinoma.

## 2 Material and Methods

### 2.1 Materials

Cetyltrimethyl ammonium bromide (CTAB), tetraethoxysilane (TEOS), and Hoechst 33342 were purchased from Sigma-Aldrich (St Louis, United States). Indocyanine Green (ICG) and 1,1′-dioctadecyl-3,3,3′,3′-tetramethylindodicarbocyanine (DiD) were purchased from Absin (Shanghai, China). Human cervical cancer cells (Hela) were purchased from the Shanghai Institute of Cell Biology, Chinese Academy of Sciences (Shanghai, China). The membrane protein extraction kit, calcein-AM/PI Double Staining Kit, Cell Counting Kit-8 (CCK-8) and penicillin–streptomycin were purchased from Biyuntian (Jiangsu, China). Dulbecco’s modified Eagle medium (DMEM) was purchased from Gibco (Grand Island, United States). Fetal bovine serum (FBS) was purchased from Hyclone (Logan, United States). All other reagents were of analytical grade without any purification.

### 2.2 Preparation of MSNs, MSNs@ICG (MI), and MSNs@DiD (MD)

MSNs were prepared using a sol–gel method according to the previous studies with minor modification (Goel et al., 2014; Quan et al., 2015; Chaudhary et al., 2019). In brief, CTAB (0.5 g) and NaOH (0.14 g) were dissolved in distilled water (250 ml) and stirred at 80°C for 30 min. Then, the silica precursor TEOS (2.5 ml) was added dropwise to the CTAB/NaOH mixture and stirred at 80°C for 2 h. The MSNs were collected as pellets by centrifugation (20,000 g, 15 min) and washed with ethanol three times. To remove the surfactant CTAC, MSNs were extracted with a 1 wt% solution of NaCl in ethanol at 40°C for 24 h thrice. Finally, MSNs were resuspended in PBS and stored at 4°C for further use.

ICG was loaded onto the MSNs using a simple incubation method (Chaudhary et al., 2019). To maximize ICG loading of MSNs, 0.2 mg of ICG was added to the MSN suspension (1 mg/ml, 1 ml), and the mixture was stirred magnetically at room temperature for 24 h. Then, the ICG-loaded MSN nanoparticles (MSNs@ICG, MI) were collected by centrifugation (20,000 g, 15 min) and washed with PBS three times. Finally, MI was redispersed in PBS (pH 7.4).

MD was prepared for the optimization of cellular uptake. DiD was dissolved in DMSO and consequently diluted with PBS. Then, 0.2 mg of DiD was loaded onto MSNs (1 mg/ml, 1 ml) by the same incubation method mentioned before.

### 2.3 Surface Modification With Cancer Cell Membranes

Cancer cell membrane–coated MSNs@ICG (CMI) was prepared according to the previous report (Fang et al., 2020). In brief, the human cervical cancer cells, Hela, were maintained in DMEM supplemented with 10% FBS, 100  U/mL penicillin G, and 100 μg/ml streptomycin. And the cells were cultured at 37°C in a humidified atmosphere of 5% CO_2_. The cancer cell membrane (CCM) was extracted from human cervical cancer cells Hela by using a membrane protein extraction kit, following the instructions from the manufacturer (Biyuntian, China). The CCM was added to the MI dispersion, and the mixture was ultrasonically dispersed by a Scientz-IID ultrasonic homogenizer (Ningbo Scientz Biotechnology Co., Ltd., China) for 1 h. Afterward, CMI was extruded by a mini-extruder (Avanti, Canada) through the 100-nm polycarbonate membrane 20 times in the dark. The CCM coating of MSN@DiD (CCM/MSNs@DiD, CMD) was also prepared by the same procedure mentioned before.

### 2.4 Characterization of MSN-Based Nanoparticles

#### 2.4.1 The Morphology, Particle Size, and Zeta Potential of MSN-Based Nanoparticles

The morphology of MSN-based nanoparticles was observed by transmission electron microscopy (TEM). In brief, a drop of the MSN-based nanoparticle dispersion (including MSNs, MI, and CMI) was deposited onto a carbon-coated grid without negative staining. And then, the samples were dried at room temperature before examination. The TEM images of MSN-based nanoparticles were obtained by using a JEM-1400 transmission electron microscope (JEOL, Japan). The particle size and zeta potential of the MSN-based nanoparticles were measured using the dynamic light scattering method (DLS) using a Zetasizer Nano ZS90 instrument at 25°C (Malvern, United Kingdom).

#### 2.4.2 The Drug Loading and Encapsulation Efficiency

The ICG loading and encapsulation efficiency in MSNs were measured by ultraviolet–visible (UV-vis) spectrophotometry. In brief, 0.2 mg of ICG was added into the MSN suspension (1 ml, 1 mg/ml). The ICG-loaded MSNs were collected by centrifugation, and the free ICG in the supernatant was determined *via* UV-vis spectrophotometry with the maximum absorbance wavelength recorded at 806 nm. The ICG loading and encapsulation efficiency of MSNs were calculated.

#### 2.4.3 SDS-PAGE Analysis

In order to investigate the CCM coating, the SDS-PAGE gel electrophoresis assay was used for the protein characterization. In brief, samples of MSN nanoparticles were collected by centrifugation at 10, 000 g for 15 min. The mixture of samples and loading buffer with the volume ratio of 4:1 was heated to 100°C for 10 min. Afterward, the samples were performed by a 10% SDS–polyacrylamide gel and stained with Coomassie Blue.

### 2.5 Cell Cultures

Cervical cancer Hela cells were maintained in DMEM supplemented with 10% FBS, 100 U/mL penicillin, and 100 μg/ml streptomycin. Hela cells were cultured at 37°C in an incubation of the humidified atmosphere with 5% CO_2_.

### 2.6 Confocal Microscopy Study

In order to confirm the homologous targeting ability of cancer cell membrane–coated MSNs, DiD was chosen as a model fluorescent dye, and cellular uptake of CMD with different concentrations of DiD was visualized by confocal microscopy (Zeiss, Germany). In brief, Hela cells (1×10^5^ cells/well) were seeded in 6-well plates and then cultured for 24 h. Hela cells were exposed to CMD with various DiD concentrations (1.25, 2.5, 5, and 10 μg/ml), respectively. After a 12-h incubation, the treated Hela cells were washed three times with cold PBS. And, cell nuclei were stained with Hoechst 33342 for 15 min. The cellular uptake images were visualized by a Zeiss LSM 880 confocal laser scanning microscope (Zeiss, Germany).

### 2.7 *In vitro* Cytotoxicity Study

#### 2.7.1 Live/Dead Staining

The *in vitro* cytotoxicity of MSN-based nanoparticles was evaluated by live/dead staining on Hela cells. Hela cells (at a density of 1×10^5^ cells per well) were seeded into 24-well plates and cultured for 24 h. Then Hela cells were exposed to MSNs (65 μg/ml, equivalent to that in CMI without ICG), free ICG, MI, and CMI (with a final ICG concentration of 10 μg/ml) for 48 h. Afterward, the cells were stained with a calcein-AM/PI Double Staining Kit (Biyuntian, China). And the treated Hela cells were photographed under an inverted fluorescent microscope (Leica, Japan). Untreated Hela cells were used as a control.

#### 2.7.2 Cell Viability Analysis

The *in vitro* cytotoxicity of MSN-based nanoparticles on Hela cells was carried out *via* the Cell Counting Kit-8 (CCK-8) assay. Hela cells (1×10^4^ cells per well) were seeded into 96-well plates and cultured for 24 h. Then Hela cells were exposed to fresh media containing ICG or MSN nanoparticles at various concentrations. After 48-h incubation, 20 ml of CCK-8 (5 mg/ml) was added into each well, and the cells were incubated for another 4 h. Afterward, the absorbance of each well was determined *via* a microplate reader (Thermo, United States) at a wavelength of 450 nm. The cell viabilities of the MSN-based nanoparticles were calculated. The cell viability of the untreated group was chosen as the negative control.

### 2.8 *In vivo* PA Imaging

Six-week-old female BALB/C nude mice (20 g weight) were purchased from the Shanghai SLAC Laboratory Animal Co. Ltd (Shanghai, China). All the animal experiments were approved by the Institutional Animal Care and Use Committee of Guangzhou Medical University.

*In vivo* PA imaging was studied on the Hela tumor-bearing mice. In brief, 2×10^6^ Hela cells per mouse were transplanted subcutaneously into the right flanks of female nude mice. When the tumor volume reached about 100 mm^3^, the mice were randomized into three groups.

In PA imaging, the mice were anesthetized with 5% isoflurane. Then, free ICG, MI, and CMI were intravenously injected *via* the tail vein with an ICG dose of 10 mg/kg, (*n* = 3/group) (Jiang et al., 2015; Ma et al., 2021). The real-time tumor PA imaging and PA signal of free ICG and ICG formulations were recorded at 48 h postinjection using the Vevo LAZR-X multimode imaging system.

In order to further confirm the homologous targeting ability, PA imaging was also carried out *via* the Hela tumor-bearing mice model with the tumor cut in half or completely, followed by treatment with free ICG, MI, and CMI. In brief, for the mice with the tumor cut in half, the Hela tumor-bearing mice were anesthetized with 5% isoflurane, and then the tumor in mice was partially excised. And for the mice with the tumor cut completely, the mice were anesthetized with 5% isoflurane, then the tumors in mice were completely resected. The treated mice were injected intravenously with free ICG, MI, and CMI *via* the tail vein at an ICG dose of 10 mg/kg (*n* = 3/group). The real-time tumor PA imaging and PA signal of free ICG and ICG formulations were recorded at 48 h postinjection.

### 2.9 Statistical Analysis

All the results were expressed as mean ± SD. And the statistical analysis was performed in SPSS 13.0 software (SPSS, United States) *via* one-way ANOVA with Tukey’s post hoc test. A *p* value < 0.05 was considered statistically significant.

## 3 Results and Discussion

### 3.1 Preparation and Physical Characterization of CMI

**FIGURE 1:**
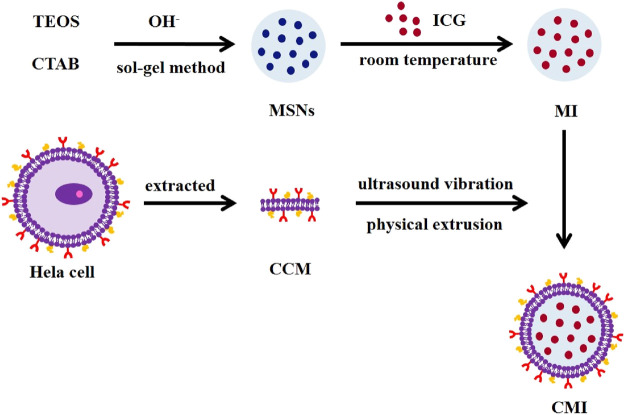
Schematic illustration of CMI.

The preparation of CMI is shown in Figure 1. MSNs were prepared by a sol–gel method, in which the surfactant CTAC was used as a template and TEOS was chosen as a silica precursor. Then, ICG was loaded onto the MSNs by a simple incubation method. To construct CMI, human cervical cancer cells Hela as the cancer cell membrane (CCM) was extracted. And the CCM was coated onto the surface of MI by ultrasound vibration and physical extrusion.

**FIGURE 2:**
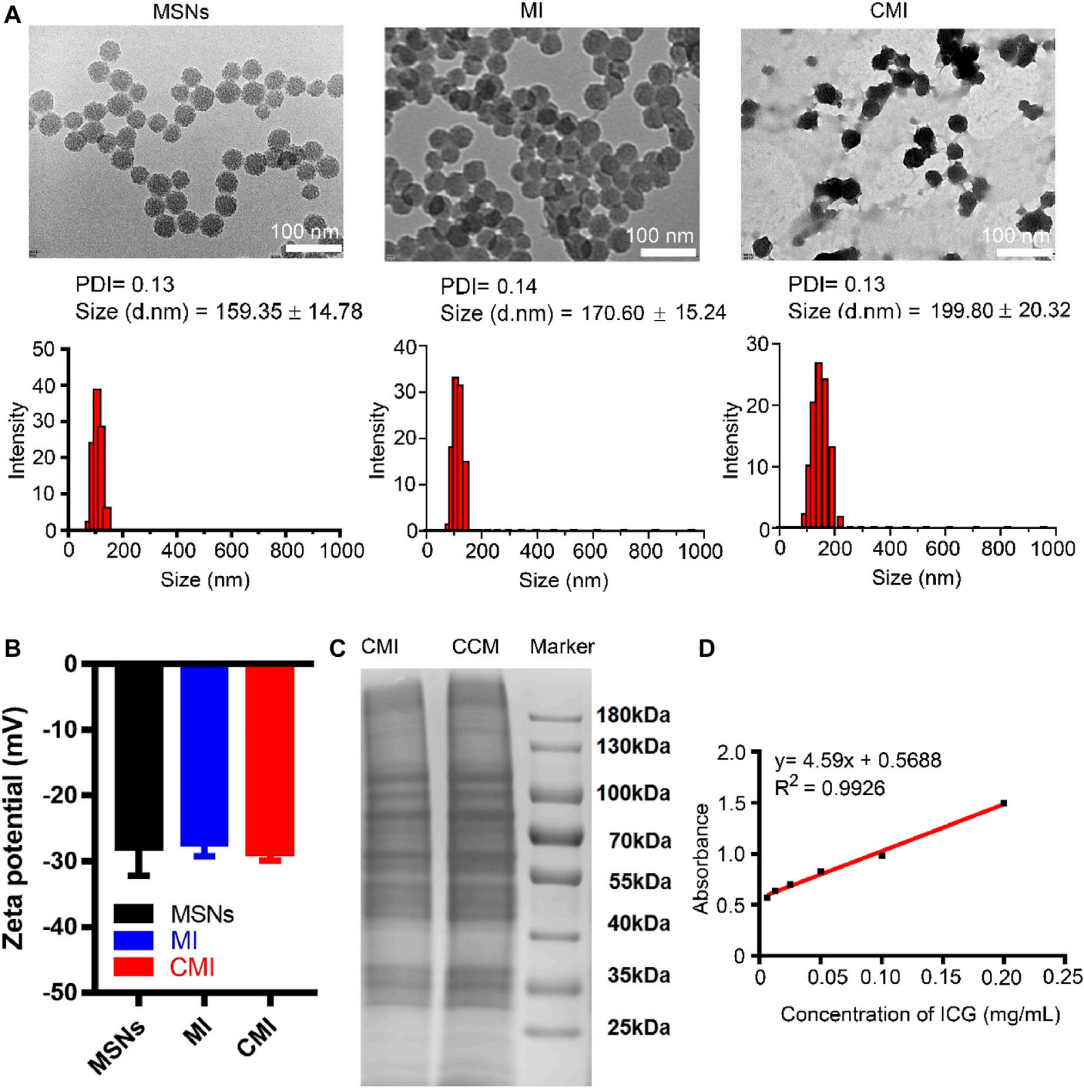
Characterization of MSN-based nanoparticles. **(A)** TEM images and particle sizes of MSN based nanoparticles. Scale bar is 100 nm in the TEM images. Particle sizes were also measured by DLS. **(B)** Zeta potential of the MSN-based nanoparticles. **(C)** SDS-PAGE analysis of MSN-based nanoparticles with 10% native polyacrylamide separating gel. **(D)** Standard curve of ICG *via* the UV-vis method.

The TEM images showed that MSNs, MI, and CMI display spherical shape with a diameter of approximately 50 nm (Figure 2A). As measured by dynamic light scattering (DLS), the hydrodynamic size of the MSNs was 159 ± 15 nm with a polydispersity index (PDI) of 0.13 (Figure 2B). Similarly, the hydrodynamic size of MI was 171 ± 15 nm, with a PDI of 0.14, which indicated that the ICG loading has no significant effect on the MSN particle size (Figure 2B). After CMM coating, the particle sizes of CMI were approximately 200 nm, with a PDI less than 0.15 (Figure 1A). The particle size of MSNs has a significant impact on the passive targeting efficiency of tumors. Mesoporous silica nanoparticles, whose particle size is smaller than 300 nm, might enhance a significant tumor accumulation *via* the EPR effect (Wang et al., 2015). The prepared CMI was small with narrow size distribution and good dispersion. These results indicated that CMI might be a promising nanoprobe for passive tumor targeting and PA imaging. The zeta potential of MSNs, MI, and CMI was approximately −30 mV (Figure 1B), which might also display good stability and long circulation characteristics for PA imaging (Cheng et al., 2020; Fang et al., 2020; Li et al., 2020). ICG possessed slight negative charges (Lajunen et al., 2018). Therefore, the driven force of MI might be mainly affected by the negative-charged MSNs. Previous reports indicated that the steric hindrance of the glycosylated domain produced the driving forces and guided the correct protein right-side-out orientation by the reduced energetic profile (Hu et al., 2014; Lin et al., 2021). Herein, CMI showed similar zeta potential to MI after CCM coating. To evaluate the stability, the particle sizes of MI and CMI were determined by DLS. CMI showed higher stability within 72 h than MI, which might be beneficial with the CCM coating.

To confirm the cell membrane modification on MSNs, the SDS-PAGE assay was carried out. The SDS-PAGE analysis showed that CMI presented a protein profile very similar to that of Hela cell membrane lysates, indicating that the cell membrane was successfully coated onto the surface of MSNs (Figure 2C). The standard curve of ICG was measured using the UV-vis method (Figure 1D). The ICG encapsulation efficiency of MSNs was about 76%, and the ICG loading efficiency was about 13% when ICG concentration was 0.2 mg/ml (Supplementary Figure S2).

### 3.2 Cellular Uptakes of the CMD

It was expected that the CCM coating from Hela cells would endow the homologous targeting ability to cervical cancer cells. To investigate the homologous targeting ability of CMI, cellular uptakes of CMI were observed in Hela cells *via* confocal microscopy. Considering that ICG is an NIR dye which effectively absorbs NIR wavelength of about 800 nm and might not be suitable for confocal microscopy, DiD was chosen as a fluorescent probe instead. Cellular uptake of the CMD with different DiD concentrations was visualized by confocal microscopy.

**FIGURE 3:**
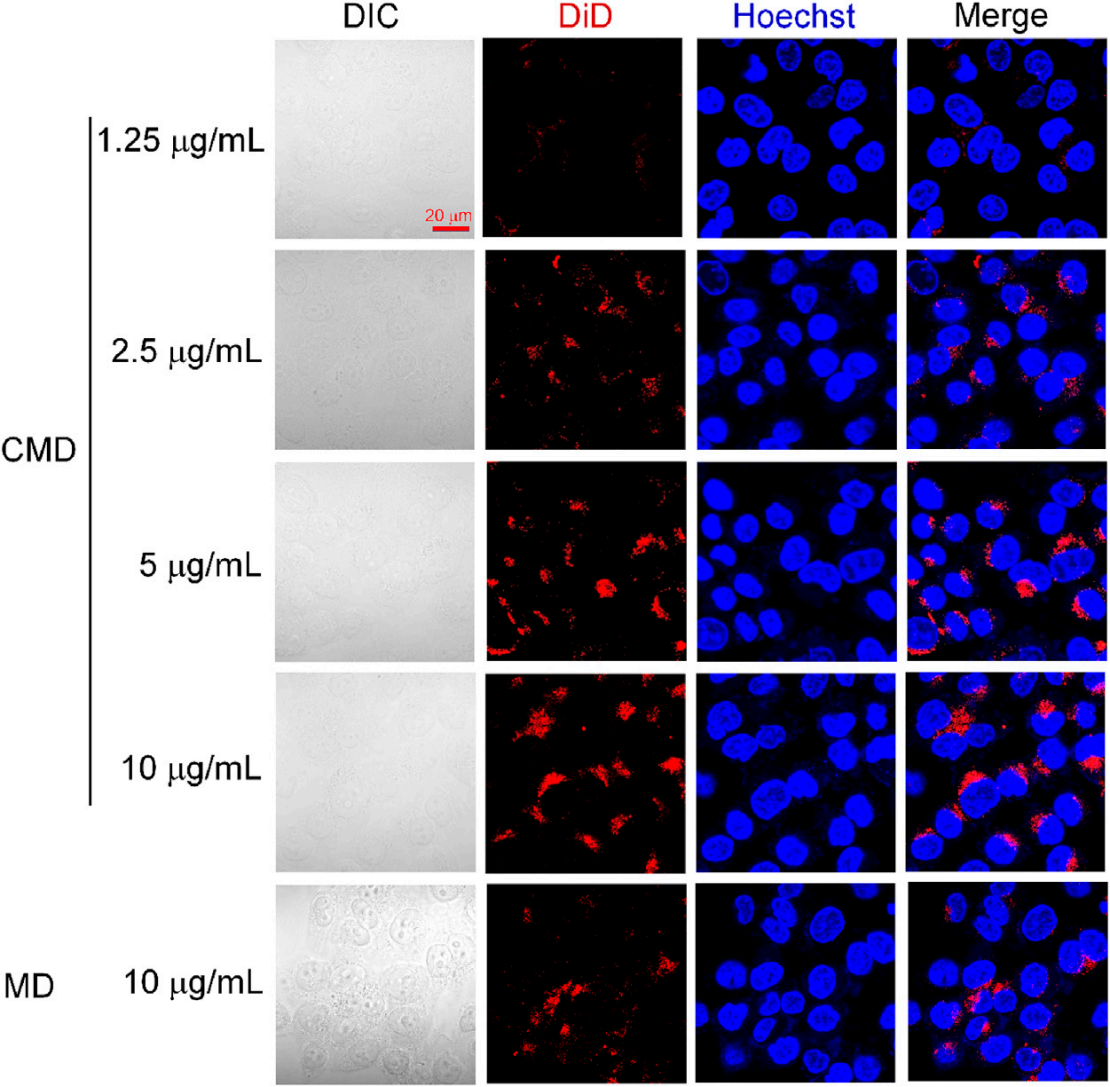
Confocal microscopy images of Hela cells treated with the CMD at different DiD concentrations. Cell nuclei were stained blue with Hoechst.

As shown in Figure 3, the DiD fluorescent intensity was found in Hela cells but not overlaid on the Hoechst nucleic acid staining, which indicated that the CMD might be distributed in the cytoplasm of Hela cells after internalization. Furthermore, with the CMD concentration increased, the DiD fluorescence intensity of Hela cells was found to be increased. The phenomenon demonstrated that the cellular uptake of the CMD in Hela cells exhibited a CMD concentration–dependent behavior. The cellular uptake of the CMD showed the most significant fluorescent intensity when DiD concentration reached upto 10 μg/ml. However, the bare nanoparticles without the CM showed lesser DiD signals (Figure 3). The confocal microscopy images indicated that the CMD might contribute the homologous targeting ability to cervical cancer Hela cells with CCM coating (Xu et al., 2020). To further confirm the homologous targeting ability, *in vivo* PA imaging should be carried out *via* Hela tumor-bearing mice with the tumor cut in half or completely.

### 3.3 *In vitro* Cytotoxicity

The fluorescent nanoprobe for application should be biocompatibility and low toxicity. In order to confirm the biocompatibility and cytotoxicity of CMI, calcein-AM/PI staining and CCK-8 were carried out. As mentioned before, the results in the confocal imaging showed that the cellular uptake of the CMD in Hela cells exhibited a CMD concentration–dependent behavior. And at 10 μg/ml DiD, the cellular uptake was obviously observed. Therefore, calcein-AM/PI staining was carried out with an ICG concentration of 10 μg/ml, in which the MSN concentration was equivalent with that of the CMD. And the ICG concentration ranging from 0 to 10 μg/ml was performed in CCK-8.

**FIGURE 4:**
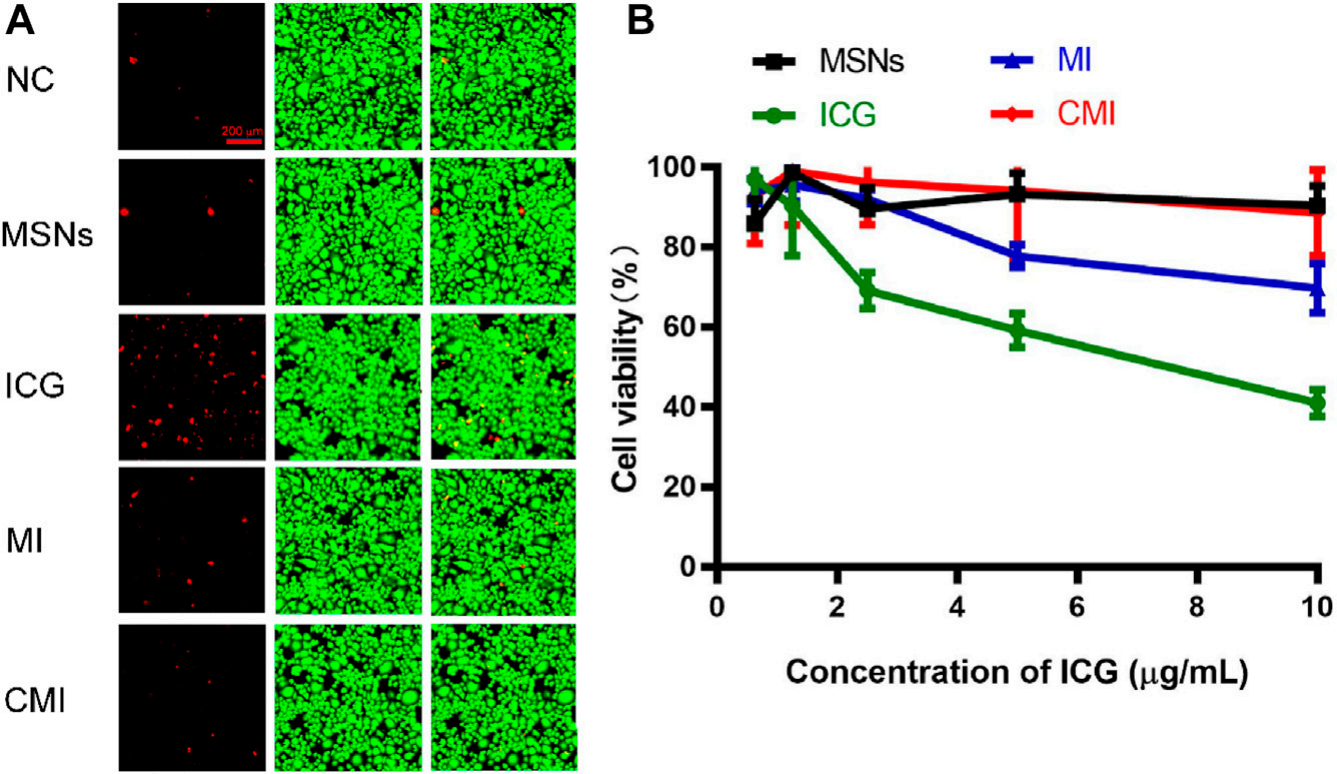
*In vitro* cytotoxicity of Hela cells treated with free ICG and MSN-based nanoparticles for 48 h. **(A)** Live/dead assay of Hela cells treated with different nanoparticles at an ICG concentration of 10 μg/ml. **(B)** CCK-8 assay of Hela cells treated with MSN-based nanoparticles with different ICG concentrations. Data are represented as mean ± SD (*n* = 3).

To confirm the biocompatibility of CMI, Hela cells exposed to CMI were stained with calcein-AM/PI for live (green) and dead (red) cell imaging, respectively (He et al., 2020). And the fluorescent images are shown in Figure 4A. Both strong green fluorescent signals and weak red fluorescent signals in Hela cells could be observed after different treatments. And the green and red fluorescent signals of MSNs, MI, and CMI were similar to those of the negative control. These results showed that Hela cell viabilities were not significantly altered after 48-h incubation with MSNs, MI and CMI. However, the red fluorescent signals of free ICG were stronger than those of the negative control and the MSN-based nanoparticles. The fluorescent images indicated that both free ICG and MSN-based nanoparticles showed no apparent toxicity in 48 h. The biocompatibility of MSN-based nanoparticles might be better than that of free ICG.

The *in vitro* cytotoxicity of ICG and MSN-based nanoparticles was also evaluated using the CCK-8 assay. The cell viabilities of free ICG and MSN-based nanoparticles on Hela cells for 48 h are represented in Figure 4B. The cell viability of MSNs without ICG was above 90%, which indicated that MSNs exhibited good biocompatibility for nanoprobe delivery. The cell viabilities of free ICG declined from 96 ± 4% to 41 ± 3% as the ICG concentration increased to 10 μg/ml. Meanwhile, at the same ICG concentration of 10 μg/ml, the cell viabilities of MI and CMI were 70 ± 6% and 90 ± 2%, respectively. The CCK-8 results were shown to be consistent with those obtained in the calcein-AM/PI dual staining assay.

The cellular uptake and intracellular drug release behaviors in Hela cells from free ICG and CMI were different (Li et al., 2017). In brief, free ICG was a small-molecule probe, with a molecular weight of 774, which entered Hela cells with passive diffusion and was directly distributed in the cellular cytoplasm. CMIs were nanoparticles with a diameter of approximately 200 nm, which entered the Hela cells with cellular uptake and then escaped from endo-/lysosomes into the cytoplasm. Finally, ICG was distributed in the cellular cytoplasm after release from the pores of CMI. It might take more time for ICG to be located in the cytoplasm from the MSNs than that without encapsulation. Therefore, the results from live/dead staining and CCK-8 assay revealed that the cytotoxicity of free ICG was the highest and that of CMI was the lowest in all the ICG formulations, which might imply that the MSN loading and CCM modification might improve the biosafety of ICG.

### 3.4 *In vivo* PA Imaging

In this study, CMI was supposed to exhibit the homologous targeting ability to Hela cancer cells *in vivo* and could be used as a fluorescent nanoprobe of PA imaging for cervical cancer. To verify the PA imaging effect, *in vivo* PA imaging of CMI on the Hela tumor-bearing mice was evaluated, and the PA signals were obtained after intravenous injection.

**FIGURE 5:**
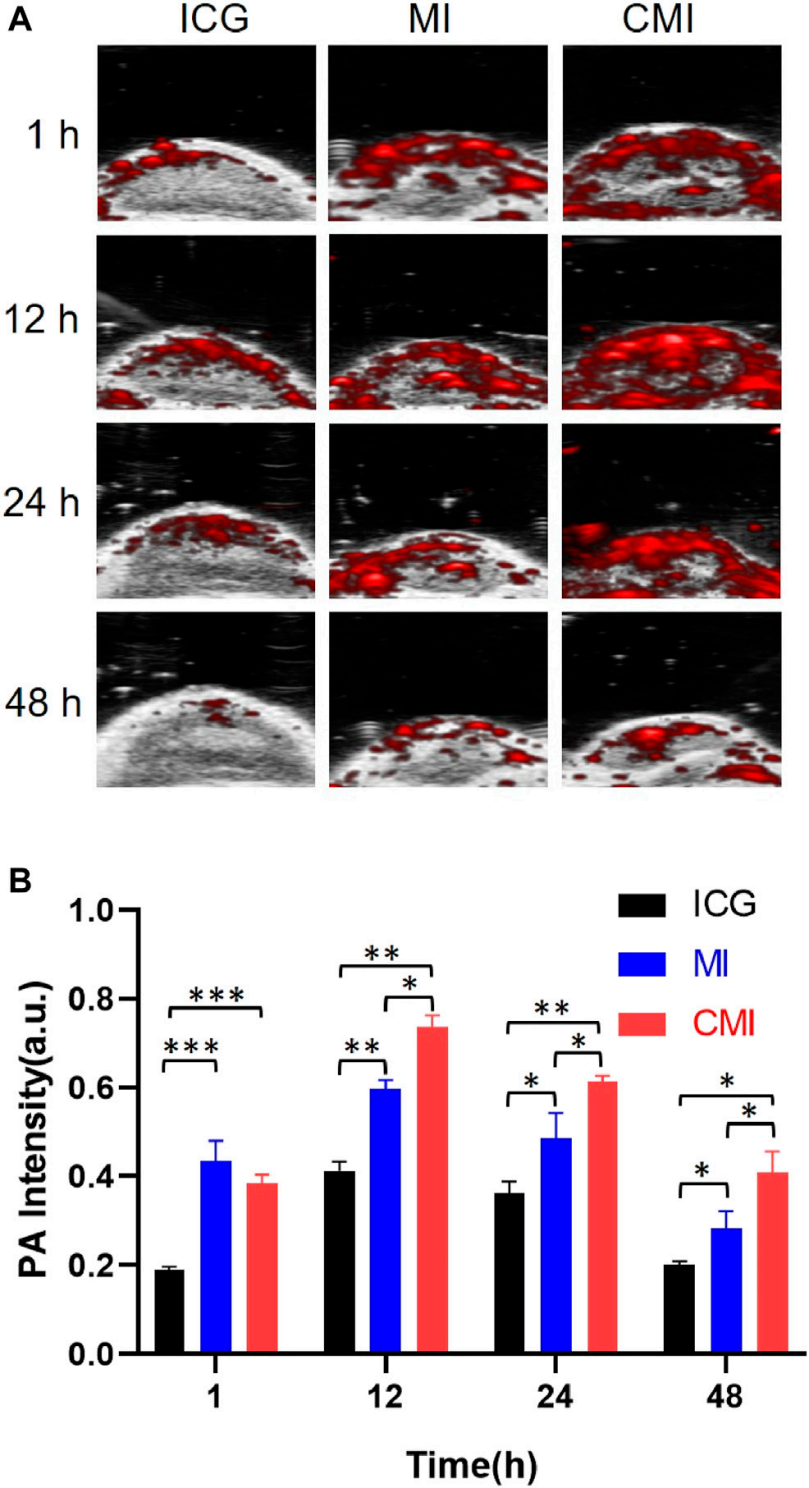
*In vivo* PA imaging of Hela tumor-bearing nude mice. **(A)** PA images of the tumor region after intravenous tail injection of ICG formulations (10 mg/kg ICG) at different time points. **(B)** Fluorescent signal measurement of ICG formulations in the tumor region at different time points. ICG, MI, and CMI (containing an ICG dose of 10 mg/kg) were injected into the Hela tumor-bearing nude mice through the tail vein (*n* = 3/group). At 1, 12, 24, and 48 h after injection, the fluorescent signals were measured by using the Step and Shoot modes with 100 angles and 15 pulses per angle in the V evo LAZR-X multimode imaging system. The results were processed by V evo LAB 3.2.0 software (*<0.05; **<0.01; ***<0.001.)

It is reported that ICG with an LD50 value of 50–80 mg/kg exhibits low toxicity for animals (http://www.drugs.com/pro/indocyanine-green.html). Doses of ICG ranging from 0 to 10 mg/kg were used to test NIR imaging by syngeneic murine flank tumor models. And it was found that the fluorescence was optimal for NIR imaging at 5 mg/kg to 10 mg/kg for 24 h (Jiang et al., 2015). 10 mg/kg ICG was applied for NIR fluorescence imaging of cervical cancer for 48 h (Ma et al., 2021). Therefore, a dose of 10 mg/kg ICG was chosen in our *in vivo* PA imaging for 48 h. As shown in Figure 5A, in the CMI group, high-fluorescence signals were easily observed in the tumor sites. The PA intensities gradually increased in the tumor site, then reached its maximum at 12 h, and finally persisted up to 48 h after injection. Further semiquantitative analysis (Figure 5B) showed that the PA intensities of both MI and CMI in the tumor were significantly stronger than those of free ICG. The PA intensity of CMI in the tumor site was 76% at 12 h, which was 0.9-fold higher (*p* < 0.01) than that of free ICG and 1/3-fold higher (*p* < 0.05) than that of MI. Results showed that CMI had stronger PA intensity in the tumor site than MI and free ICG, implying that CMI could specifically accumulate into the cervical tumor, which might be attributed to the homologous targeting ability.

**FIGURE 6:**
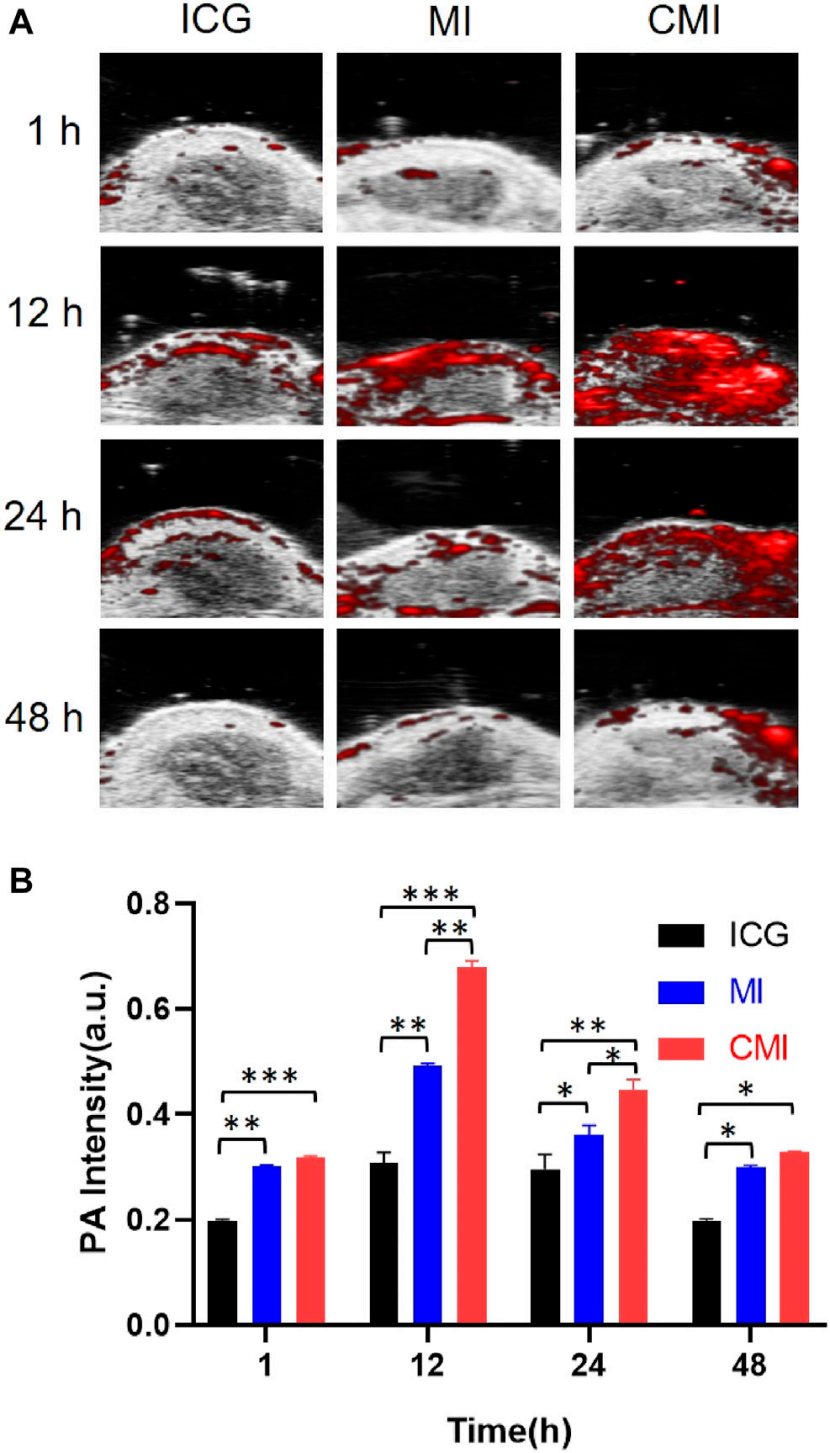
*In vivo* PA imaging of Hela tumor–bearing nude mice. **(A)** PA images of the tumor region. **(B)** Fluorescent signal measurement of the tumor region. The tumors were cut in half, followed by treatment with ICG, MI, or CMI (containing an ICG dose of 10 mg/kg) injected into the Hela tumor-bearing nude mice through the tail vein (n = 3/group). At 1, 12, 24, and 48 h after injection, the fluorescent signals were measured by using the Step and Shoot mode with 100 angles and 15 pulses per angle in the V evo LAZR-X multimode imaging system. The results were processed by V evo LAB 3.2.0 software (*<0.05; **<0.01; ***<0.001.)

**FIGURE 7:**
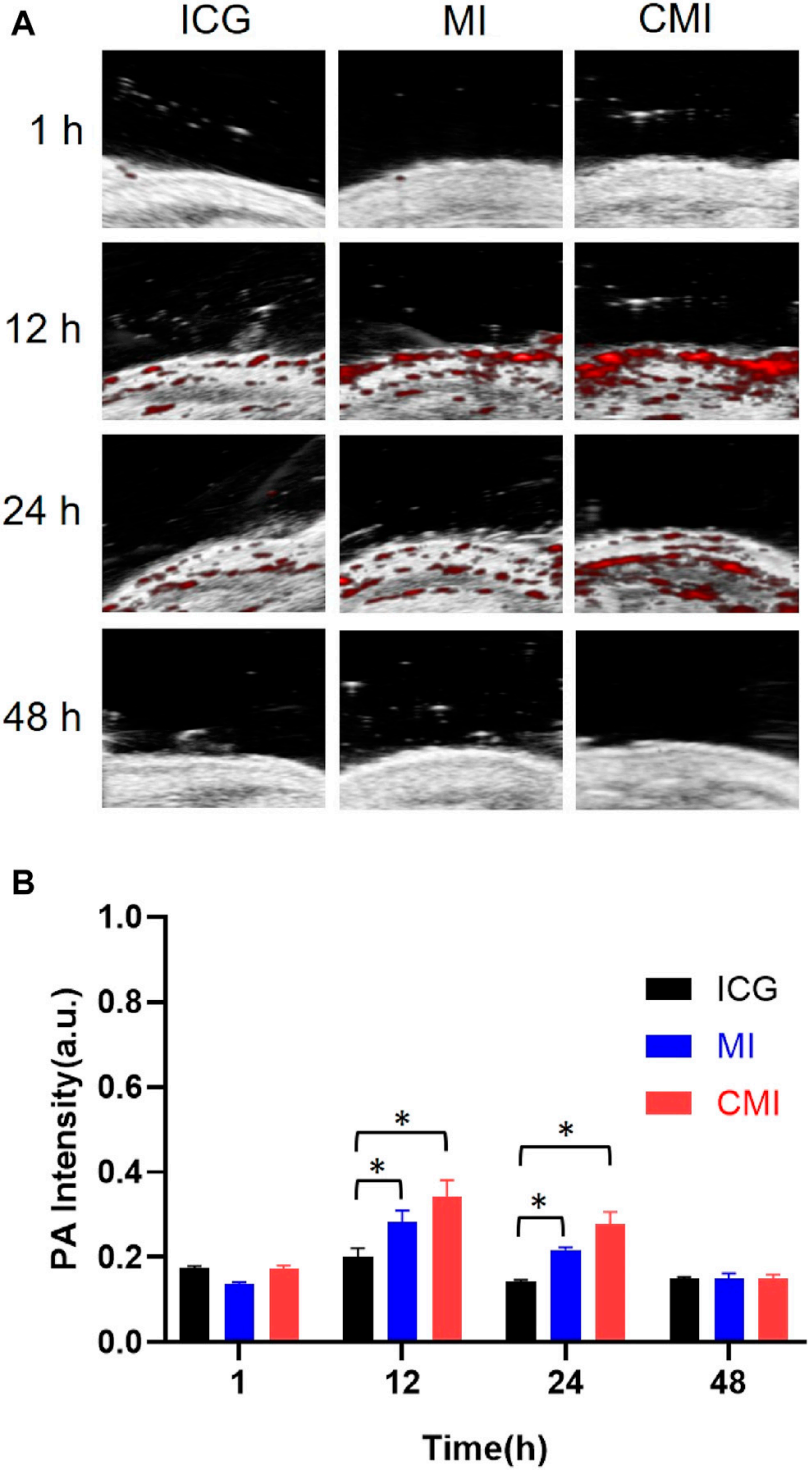
*In vivo* PA imaging of Hela tumor–bearing nude mice. **(A)** PA images of the tumor region. **(B)** Fluorescent signal measurement of ICG formulations in the tumor region. The tumors were cut completely, followed by treatment with ICG, MI, and CMI (containing an ICG dose of 10 mg/kg) *via* the tail vein (*n* = 3/group). At 1, 12, 24, and 48 h after injection, the fluorescent signals were measured by using the Step and Shoot mode with 100 angles and 15 pulses per angle in the V evo LAZR-X multimode imaging system. The results were processed by V evo LAB 3.2.0 software (*<0.05).

According to the homologous targeting ability, CMI could show enhanced PA imaging on the cervical tumor, but the enhanced PA imaging might disappear after complete tumor resection. In order to further confirm the homologous targeting ability, in the following PA imaging experiments, the tumors were cut in half or completely in the mice, followed by treatment with free ICG, MI, and CMI. We found that the PA signals in the half tumors treated with CMI were also much higher than free ICG or MI-treated ones (Figures 6A,B). However, after the tumors were excised, the PA signals in the CMI-treated group recovered to the free ICG or MI level (Figures 7A,B). Interestingly, the *in vivo* PA intensities of CMI and MI were significantly higher than those of ICG at 12 and 24 h (*p* < 0.05). But at the same time, the PA intensities of CMI and MI had no significant differences (*p* > 0.05). The phenomena might be attributed to the MSN encapsulation, which could prolong the circulation of ICG. For PA imaging, free ICG was rapidly cleared *in vivo*; therefore, the ICG distribution was limited, and the ICG signal was low after 12 h. When encapsulated into the MSNs, ICG was protected from the reticuloendothelial system by MSNs; therefore, the body clearance of ICG was slowed down, and the ICG signal from CMI and MI was higher than free ICG after tumor resection.

These results showed that CMI displayed the strongest PA intensity in the tumor site, demonstrating that CMI could specifically accumulate into the tumor. The superior PA imaging efficacy of CMI might be attributed to the EPR effect *via* MSN loading and homologous binding ability *via* CCM coating (Peng et al., 2021).

In our present study, Hela cell membrane coating MSNs was constructed for the homotypic cancer diagnosis. All the results showed that CMI might be a favorable biomimetic nanoprobe for PA imaging, which might be benefited from the MSN loading and Hela cell membrane coating. First, owing to the special structure of MSNs, ICG was successfully loaded into the pore of MSN. And the nanostructure of MSNs could improve the photostability and prolong the circulation of ICG. Besides, MSNs with particle sizes less than 300 nm were endowed with passive targeting ability *via* the EFR effect. Second, owing to Hela cell membrane coating, CMI was endowed with homologous binding ability to cervical carcinoma, which efficiently delivered ICG to cervical cancer *via* the homotypic recognition to the same cell lines. Herein, the enhanced PA imaging of cervical cancer was achieved *via* MSN loading and Hela cell membrane coating.

PA imaging is a new non-invasive imaging technology that combines with ultrasonic detection and optical excitation. The detected tissue acoustic waves which are emitted by the pulsed laser excitation can be detected by an ultrasound transducer, and the PA images are reconstructed by the absorbed optical energy distribution (Zeng et al., 2018). The PA images are locally imaged that generally focus on the detected tissue, which is different from the NIR imaging that could provide with body imaging (Zeng et al., 2018; Chaudhary et al., 2019). In our study, the biomimetic probe CMI was applied to PA imaging on cervical cancer, so the PA imaging focused on cervical tumor sites, and the PA images on cervical tumors were provided. The PA imaging of CMI displayed enhanced PA intensity on the cervical tumor sites compared with free ICG and MI. However, no directing results could be sure whether CMI was penetrated into the tumor or remained on the tumor surface, CMI could improve the fluorescence intensity on the cervical tumor site, which might be beneficial for PA imaging *via* the homologous targeting ability. Our results showed that CMI could be a promising probe for PA imaging of cervical tumor tissues. And, the multimodality precise diagnosis combined with PA/NIR imaging and photothermal therapy would be performed further, and the total body images and tumor inhibitory effects will be discussed in our future research.

## 4 Conclusion

In summary, a novel biomimetic nanoprobe cancer membrane–coated MSN loaded with ICG (CMI) was successfully developed for PA imaging of cervical carcinoma. The nanoprobe CMI with small particle size exhibited large loading efficiency, good biocompatibility and low toxicity. Furthermore, it could specifically accumulate into the tumor, which displayed a superior PA imaging efficacy that allowed for non-invasive deep tissue imaging of cervical carcinoma *in vivo*. Therefore, CMI might be a potential fluorescent probe for PA imaging of cervical carcinoma.

## Data Availability

The original contributions presented in the study are included in the article/Supplementary Material; further inquiries can be directed to the corresponding authors.

## References

[B1] Arbyn M. Weiderpass E. Bruni L. de Sanjosé S. Saraiya M. Ferlay J. (2020). Estimates of Incidence and Mortality of Cervical Cancer in 2018: a Worldwide Analysis. Lancet Glob. Health 8 (2), e191–e203. 10.1016/S2214-109X(19)30482-6 31812369 PMC7025157

[B2] Chaudhary Z. Khan G. M. Abeer M. M. Pujara N. Wan-Chi Tse B. McGuckin M. A. (2019). Efficient Photoacoustic Imaging Using Indocyanine green (ICG) Loaded Functionalized Mesoporous Silica Nanoparticles. Biomater. Sci. 7 (12), 5002–5015. 10.1039/c9bm00822e 31617526

[B3] Cheng Y.-J. Hu J.-J. Qin S.-Y. Zhang A.-Q. Zhang X.-Z. (2020). Recent Advances in Functional Mesoporous Silica-Based Nanoplatforms for Combinational Photo-Chemotherapy of Cancer. Biomaterials 232, 119738. 10.1016/j.biomaterials.2019.119738 31901695

[B4] Croissant J. G. Zhang D. Alsaiari S. Lu J. Deng L. Tamanoi F. (2016). Protein-gold Clusters-Capped Mesoporous Silica Nanoparticles for High Drug Loading, Autonomous Gemcitabine/doxorubicin Co-delivery, and *In-Vivo* Tumor Imaging. J. Controlled Release 229, 183–191. 10.1016/j.jconrel.2016.03.030 27016140

[B5] Defteralı Ç. Verdejo R. Majeed S. Boschetti-de-Fierro A. Méndez-Gómez H. R. Díaz-Guerra E. (2016). *In Vitro* Evaluation of Biocompatibility of Uncoated Thermally Reduced Graphene and Carbon Nanotube-Loaded PVDF Membranes with Adult Neural Stem Cell-Derived Neurons and Glia. Front. Bioeng. Biotechnol. 4, 94. 10.3389/fbioe.2016.00094 27999773 PMC5138223

[B6] Dehaini D. Wei X. Fang R. H. Masson S. Angsantikul P. Luk B. T. (2017). Erythrocyte-platelet Hybrid Membrane Coating for Enhanced Nanoparticle Functionalization. Adv. Mater. 29 (16), 1606209. 10.1002/adma.201606209 PMC5469720 28199033

[B7] Fang H. Li M. Liu Q. Gai Y. Yuan L. Wang S. (2020). Ultra-sensitive Nanoprobe Modified with Tumor Cell Membrane for UCL/MRI/PET Multimodality Precise Imaging of Triple-Negative Breast Cancer. Nano-micro Lett. 12 (1), 62. 10.1007/s40820-020-0396-4 PMC7770711 34138297

[B8] Fang R. H. Kroll A. V. Gao W. Zhang L. (2018). Cell Membrane Coating Nanotechnology. Adv. Mater. 30 (23), 1706759. 10.1002/adma.201706759 PMC5984176 29582476

[B9] Gao C. Dong P. Lin Z. Guo X. Jiang B.-P. Ji S. (2018). Near-Infrared Light Responsive Imaging-Guided Photothermal and Photodynamic Synergistic Therapy Nanoplatform Based on Carbon Nanohorns for Efficient Cancer Treatment. Chem. Eur. J. 24 (49), 12827–12837. 10.1002./chem.20180261110.1002/chem.201802611 29978545

[B10] Gao Y. Gao D. Shen J. Wang Q. (2020). A Review of Mesoporous Silica Nanoparticle Delivery Systems in Chemo-Based Combination Cancer Therapies. Front. Chem. 8, 598722. 10.3389/fchem.2020.598722 33330389 PMC7732422

[B11] Goel S. Chen F. Hong H. Valdovinos H. F. Hernandez R. Shi S. (2014). VEGF121-conjugated Mesoporous Silica Nanoparticle: a Tumor Targeted Drug Delivery System. ACS Appl. Mater. Inter. 6 (23), 21677–21685. 10.1021/am506849p PMC4262629 25353068

[B12] He Y.-J. Liu X.-Y. Xing L. Wan X. Chang X. Jiang H.-L. (2020). Fenton Reaction-independent Ferroptosis Therapy via Glutathione and Iron Redox Couple Sequentially Triggered Lipid Peroxide Generator. Biomaterials 241, 119911. 10.1016/j.biomaterials.2020.119911 32143060

[B13] Hu C.-M. J. Fang R. H. Luk B. T. Zhang L. (2014). Polymeric Nanotherapeutics: Clinical Development and Advances in Stealth Functionalization Strategies. Nanoscale 6, 65–75. 10.1039/c3nr05444f 24280870

[B14] Huang G. Su C. Wang L. Fei Y. Yang J. (2021). The Application of Nucleic Acid Probe-Based Fluorescent Sensing and Imaging in Cancer Diagnosis and Therapy. Front. Chem. 9, 705458. 10.3389/fchem.2021.705458 34141699 PMC8204288

[B15] Huang T.-Y. Huang G.-L. Zhang C.-Y. Zhuang B.-W. Liu B.-X. Su L.-Y. (2020). Supramolecular Photothermal Nanomedicine Mediated Distant Tumor Inhibition via PD-1 and TIM-3 Blockage. Front. Chem. 8, 1. 10.3389/fchem.2020.00001 32117862 PMC7034522

[B16] Jafari S. Derakhshankhah H. Alaei L. Fattahi A. Varnamkhasti B. S. Saboury A. A. (2019). Mesoporous Silica Nanoparticles for Therapeutic/diagnostic Applications. Biomed. Pharmacother. 109, 1100–1111. 10.1016/j.biopha.2018.10.167 30551360

[B17] Jiang J. X. Keating J. J. Jesus E. M. Judy R. P. Madajewski B. Venegas O. (2015). Optimization of the Enhanced Permeability and Retention Effect for Near-Infrared Imaging of Solid Tumors with Indocyanine green. Am. J. Nucl. Med. Mol. Imaging 5 (4), 390–400. 26269776 PMC4529592

[B18] Kim M. W. Lee G. Niidome T. Komohara Y. Lee R. Park Y. I. (2020). Platelet-like Gold Nanostars for Cancer Therapy: the Ability to Treat Cancer and Evade Immune Reactions. Front. Bioeng. Biotechnol. 8, 133. 10.3389/fbioe.2020.00133 32158752 PMC7051916

[B19] Koh W.-J. Abu-Rustum N. R. Bean S. Bradley K. Campos S. M. Cho K. R. (2019). Cervical Cancer, Version 3.2019, NCCN Clinical Practice Guidelines in Oncology. J. Natl. Compr. Canc Netw. 17 (1), 64–84. 10.6004/jnccn.2019.0001 30659131

[B20] Kunde S. S. Wairkar S. (2021). Platelet Membrane Camouflaged Nanoparticles: Biomimetic Architecture for Targeted Therapy. Int. J. Pharmaceutics 598, 120395. 10.1016/j.ijpharm.2021.120395 33639226

[B21] Lajunen T. Nurmi R. Wilbie D. Ruoslahti T. Johansson N. G. Korhonen O. (2018). The Effect of Light Sensitizer Localization on the Stability of Indocyanine green Liposomes. J. Controlled Release 284, 213–223. 10.1016/j.jconrel.2018.06.029 29964133

[B22] Li H. Peng Q. Yang L. Lin Y. Chen S. Qin Y. (2020). High-Performance Dual Combination Therapy for Cancer Treatment with Hybrid Membrane-Camouflaged Mesoporous Silica Gold Nanorods. ACS Appl. Mater. Inter. 12 (52), 57732–57745. 10.1021/acsami.0c18287 33326211

[B23] Li T. Qin X. Li Y. Shen X. Li S. Yang H. (2020). Cell Membrane Coated-Biomimetic Nanoplatforms toward Cancer Theranostics. Front. Bioeng. Biotechnol. 8, 371. 10.3389/fbioe.2020.00371 32411690 PMC7202082

[B24] Li Y. Liu G. Ma J. Lin J. Lin H. Su G. (2017). Chemotherapeutic Drug-Photothermal Agent Co-self-assembling Nanoparticles for Near-Infrared Fluorescence and Photoacoustic Dual-Modal Imaging-Guided Chemo-Photothermal Synergistic Therapy. J. Controlled Release 258, 95–107. 10.1016/j.jconrel.2017.05.011 28501673

[B25] Lin Y. Li S. Xiao Z. Chen S. Yang L. Peng Q. (2021). Epigenetic Inhibition Assisted Chemotherapeutic Treatment of Lung Cancer Based on Artificial Exosomes. Pharmacol. Res. 171, 105787. 10.1016/j.phrs.2021.105787 34314859

[B26] Ma R. Alifu N. Du Z. Chen S. Heng Y. Wang J. (2021). Indocyanine green-based Theranostic Nanoplatform for NIR Fluorescence Image-Guided Chemo/photothermal Therapy of Cervical Cancer. Int. J. Nanomedicine 16, 4847–4861. 10.2147/IJN.S318678 34305398 PMC8297555

[B27] Meng X. Zhang J. Sun Z. Zhou L. Deng G. Li S. (2018). Hypoxia-triggered Single Molecule Probe for High-Contrast NIR II/PA Tumor Imaging and Robust Photothermal Therapy. Theranostics 8 (21), 6025–6034. 10.7150/thno.26607 30613279 PMC6299436

[B28] Peng Q. Li H. Deng Q. Liang L. Wang F. Yang L. (2021). Hybrid Artificial Cell-Mediated Epigenetic Inhibition in Metastatic Lung Cancer. J. Colloid Interf. Sci. 603, 319–332. 10.1016/j.jcis.2021.06.066 34186407

[B29] Qiu T. Lan Y. Wei Z. Zhang Y. Lin Y. Tu C. (2021). *In Vivo* Multi-scale Photoacoustic Imaging Guided Photothermal Therapy of Cervical Cancer Based on Customized Laser System and Targeted Nanoparticles. Int. J. Nanomedicine 16, 2879–2896. 10.2147/IJN.S301664 33883896 PMC8055284

[B30] Quan G. Pan X. Wang Z. Wu Q. Li G. Dian L. (2015). Lactosaminated Mesoporous Silica Nanoparticles for Asialoglycoprotein Receptor Targeted Anticancer Drug Delivery. J. Nanobiotechnol 13, 7. 10.1186/s12951-015-0068-6 PMC4333889 25643602

[B31] Reinhardt C. J. Chan J. (2018). Development of Photoacoustic Probes for *In Vivo* Molecular Imaging. Biochemistry 57 (2), 194–199. 10.1021/acs.biochem.7b00888 29022344 PMC7875431

[B32] Sun Y. Ding F. Chen Z. Zhang R. Li C. Xu Y. (2019a). Melanin-dot-mediated Delivery of Metallacycle for NIR-II/photoacoustic Dual-Modal Imaging-Guided Chemo-Photothermal Synergistic Therapy. Proc. Natl. Acad. Sci. USA 116 (34), 16729–16735. 10.1073/pnas.1908761116 31391305 PMC6708342

[B33] Sun Y. Ding F. Zhou Z. Li C. Pu M. Xu Y. (2019b). Rhomboidal Pt(II) Metallacycle-Based NIR-II Theranostic Nanoprobe for Tumor Diagnosis and Image-Guided Therapy. Proc. Natl. Acad. Sci. USA 116 (6), 1968–1973. 10.1073/pnas.1817021116 30670648 PMC6369813

[B34] Wang Y. Zhao Q. Han N. Bai L. Li J. Liu J. (2015). Mesoporous Silica Nanoparticles in Drug Delivery and Biomedical Applications. Nanomedicine: Nanotechnology, Biol. Med. 11 (2), 313–327. 10.1016/j.nano.2014.09.014 25461284

[B35] Xu C.-H. Ye P.-J. Zhou Y.-C. He D.-X. Wei H. Yu C.-Y. (2020). Cell Membrane-Camouflaged Nanoparticles as Drug Carriers for Cancer Therapy. Acta Biomater. 105, 1–14. 10.1016/j.actbio.2020.01.036 32001369

[B36] Xu G. Bao X. Chen J. Zhang B. Li D. Zhou D. (2019). *In Vivo* tumor Photoacoustic Imaging and Photothermal Therapy Based on Supra-(carbon Nanodots). Adv. Healthc. Mater. 8 (2), 1800995. 10.1002/adhm.201800995 30474227

[B37] Yaman S. Ramachandramoorthy H. Oter G. Zhukova D. Nguyen T. Sabnani M. K. (2020). Melanoma Peptide MHC Specific TCR Expressing T-Cell Membrane Camouflaged PLGA Nanoparticles for Treatment of Melanoma Skin Cancer. Front. Bioeng. Biotechnol. 8, 943. 10.3389/fbioe.2020.00943 32850765 PMC7431670

[B38] Yang R. Xu J. Xu L. Sun X. Chen Q. Zhao Y. (2018). Cancer Cell Membrane-Coated Adjuvant Nanoparticles with Mannose Modification for Effective Anticancer Vaccination. ACS Nano 12 (6), 5121–5129. 10.1021/acsnano.7b09041 29771487

[B39] Zeng L. Ma G. Lin J. Huang P. (2018). Photoacoustic Probes for Molecular Detection: Recent Advances and Perspectives. Small 14 (30), 1800782. 10.1002/smll.201800782 29873182

[B40] Zhang L. Deng S. Zhang Y. Peng Q. Li H. Wang P. (2020a). Homotypic Targeting Delivery of siRNA with Artificial Cancer Cells. Adv. Healthc. Mater. 9 (9), 1900772. 10.1002/adhm.201900772 32181988

[B41] Zhang Y. Qin Y. Li H. Peng Q. Wang P. Yang L. (2020b). Artificial Platelets for Efficient siRNA Delivery to clear “Bad Cholesterol”. ACS Appl. Mater. Inter. 12, 28034–28046. 10.1021/acsami.0c07559 32469502

