# Supplementary material for: Efficient Photoacoustic Imaging With Biomimetic Mesoporous Silica-Based Nanoparticles
Source: Front Bioeng Biotechnol. 2021 Nov 30;9:762956. doi: 10.3389/fbioe.2021.762956 (PMC8669651; doi:10.3389/fbioe.2021.762956)
Supplement: Supplementary file 1 [file DataSheet1.PDF]

Supporting information

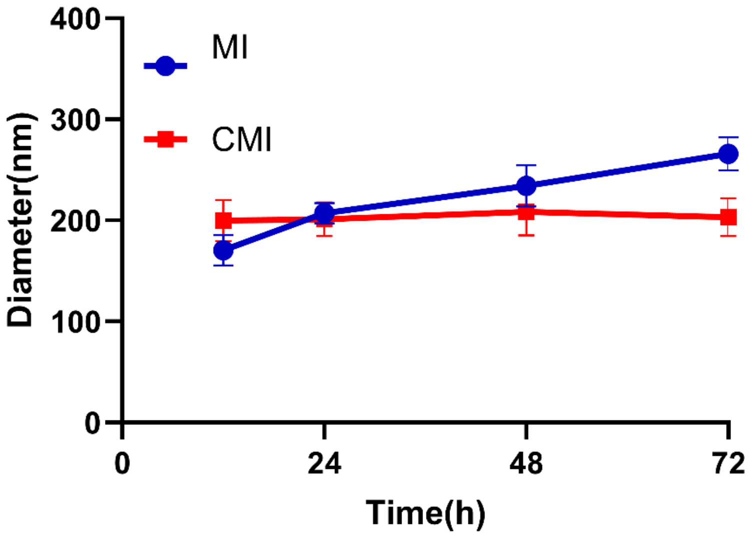

Figure S1. The stability of different nanoparticles. The size of nanoparticles was measured with DLS.

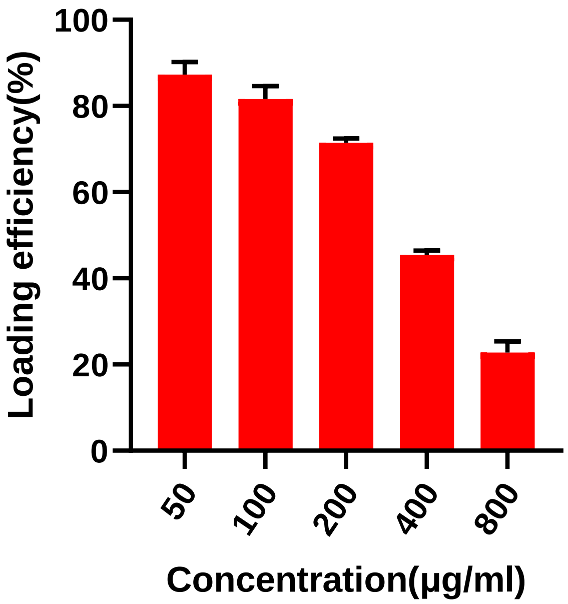

Figure S2. The loading efficiency of ICG by MSN.
